# SnoRNA in Cancer Progression, Metastasis and Immunotherapy Response

**DOI:** 10.3390/biology10080809

**Published:** 2021-08-20

**Authors:** Jildou van der Werf, Chue Vin Chin, Nicholas Ian Fleming

**Affiliations:** Department of Pathology, University of Otago, Dunedin 9016, New Zealand; jildou.vdwerf@outlook.com (J.v.d.W.); chuevin@gmail.com (C.V.C.)

**Keywords:** snoRNA, cancer, mTOR, ribosome, immunotherapy, immune checkpoint inhibitors

## Abstract

**Simple Summary:**

A much larger number of small nucleolar RNA (snoRNA) have been found encoded within our genomes than we ever expected to see. The activities of the snoRNAs were thought restricted to the nucleolus, where they were first discovered. Now, however, their significant number suggests that their functions are more diverse. Studies in cancers have shown snoRNA levels to associate with different stages of disease progression, including with metastasis. In addition, relationships between snoRNA levels and response to immunotherapies, have been reported. Emerging technologies now allow snoRNA to be targeted directly in cancers, and the therapeutic value of this is being explored.

**Abstract:**

Small nucleolar RNA (snoRNA) were one of our earliest recognised classes of non-coding RNA, but were largely ignored by cancer investigators due to an assumption that their activities were confined to the nucleolus. However, as full genome sequences have become available, many new snoRNA genes have been identified, and multiple studies have shown their functions to be diverse. The consensus now is that many snoRNA are dysregulated in cancers, are differentially expressed between cancer types, stages and metastases, and they can actively modify disease progression. In addition, the regulation of the snoRNA class is dominated by the cancer-supporting mTOR signalling pathway, and they may have particular significance to immune cell function and anti-tumour immune responses. Given the recent advent of therapeutics that can target RNA molecules, snoRNA have robust potential as drug targets, either solely or in the context of immunotherapies.

## 1. Introduction

Small nucleolar RNA (snoRNA) have a fascinating history, dating back to the very first years of RNA biology. For that same reason, they were largely ignored by cancer researchers until genome sequencing revealed that many more exist than initially expected. In a recent assessment, at least 2000 snoRNA were annotated within our human genomes [1].

Early work rightly determined that many snoRNAs function within the nucleolus, where they were first found, and that they target post-transcriptional modifications into ribosomal RNAs (rRNA). This in turn, supports the generation of robust ribosome ribonucleoprotein (RNP) complexes. However, their updated number and increased appreciation for non-coding RNA functionality has led to a reassessment of their activities. Presently, multiple reports reveal a functional diversity amongst these molecules. It is now clear that their influence extends well beyond the nucleolus into the wider nucleus and even cytoplasm. Moreover, it appears that snoRNA are impactful in health and disease, and not least in cancer biology. Recently, we have seen multiple reports that implicate these molecules in the establishment, progression and metastasis of cancers. Perhaps more importantly, we have seen that they are significant regulators in the immune system that fights against them.

## 2. SnoRNA: Long Known Regulators of Ribosomal RNA Maturation

**Figure 1:**
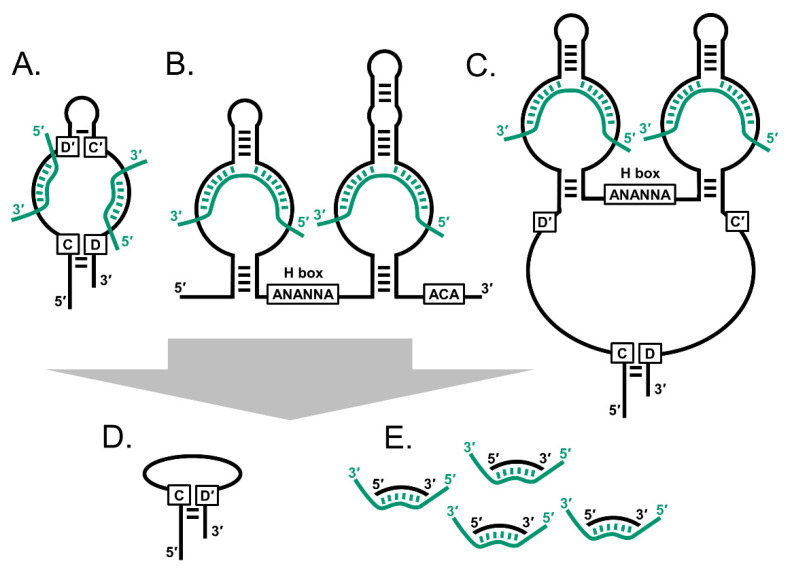
The snoRNAs are an interrelated family of small non-coding RNA. (**A**) C/D box snoRNAs (SNORDs) typically have two sets of C/D boxes that mediate hairpin structures. (**B**) H/ACA box snoRNAs (SNORAs) have an H box and ACA motifs. (**C**) Small Cajal body-specific RNAs (SCARNAs) have combinations of the two motif sets. Smaller snoRNA derived species include (**D**) C/D box-like snoRNAs and (**E**) snoRNA-derived RNAs (sdRNAs). Black strands: snoRNA; green strands: rRNA, spliceosomal snRNA and mRNA.

The name snoRNA was coined in 1982 [2], following foundation studies by a number of investigators, including the eminent cancer researcher Bob Weinberg [3]. Within the nucleus, a series of small nuclear RNAs (snRNAs) were identified as being facilitators of mRNA splicing (U1–U6), and molecules specific to nucleolus (snoRNA) were named (e.g., U3, 5.4, B1, B2, 7.1 and 7.2) [2,4]. Strictly speaking, the term snoRNA originally referred only to those small nucleolus RNAs, but in subsequent years it has evolved to encompass a small group of related RNA structures that, in many cases, operate in other nuclear structures and even the cytoplasm. The classic snoRNA’s fall into three major groups: first, the C/D box snoRNAs (SNORDs) are typically 60 to 90 nt long and contain C/D box motifs (C: RUGAUGA and D: CUGA). Next, the H/ACA box snoRNAs (SNORAs) range from 120 to 140 nt and include H/ACA box motifs (H: ANANNA and ACA: ACA) [5]. Third, the small Cajal body-specific RNAs (SCARNAs) are more variable in length and include the C/D and H/ACA motifs in varying combinations (Figure 1). The SNORDs and SNORAs typically reside in the nucleolus where they are incorporated into RNPs that modify rRNA, and the SCARNAs localise to Cajal bodies where they are thought to help modify U1 to U6 [6]. However, a growing list of variants have emerged, and currently, the snoRNA class should be considered a spectrum of molecules incorporating these motifs [7] (Figure 1). For example, Chen et al. recently described long non-coding RNA (lncRNA) species that use C/D box structures as stabilisation alternatives to 5′caps and polyadenylation [8,9,10]. Further, a growing series of reports indicate that these molecules are subject to significant additional processing so that multiple smaller snoRNA-derived RNAs exist (sdRNAs), sometimes trimmed down to discrete C/D boxes [11] or instead down to sizes similar to microRNA (miRNA) and piwi-interacting RNA (piRNA) [12,13].

Functionally, the C/D box is essential for SNORD snoRNAs to form a hairpin loop and interact with the RNP proteins NOP58, NOP56 and SNu13, together with the RNA methyl transferase fibrillarin (FBL), which then post-transcriptionally methylate rRNA at sites that complement sequences within the snoRNA [14,15]. Likewise, H/ACA box structures enable SNORA molecules to form RNPs with NOP10, NHP2, GAR1 and dyskerin pseudouridine synthase 1 (DKC1), as well as to induce pseudouridation in rRNAs using complementarity to the snoRNA [16,17] (Figure 1). In the human genome, snoRNA genes are generally encoded within introns of larger host genes, either specifying proteins or larger lncRNA, which in some cases appear dedicated to the purpose. Well known examples include growth arrest-specific transcript 5 (GAS5), ZNFX1 antisense RNA 1 (ZFAS1) and a series of at least 27 genes named small nucleolar RNA host genes 1–32 (SNHG1-32). However, the most exciting aspects of the snoRNA field is that there are many more snoRNA genes than previously thought, and the list of processes they influence is expanding. Recent efforts to consolidate records for these (e.g., RefSeq, Ensembl, RNAcentral, Rfam, snOPY) determined that we have at least 461 identifiable unique SNORD genes, 246 SNORAs and 21 SCARNAs within our genomes. Due to gene duplication, there is at least 2064 separate snoRNA genes [1,7]. Significantly, there appear to be many more snoRNA than those required to modify known nuclear targets such as rRNA, and more than half have no elucidated function, i.e., they are “orphan snoRNA”.

## 3. The Wider Biological Activities of snoRNA Extend Well beyond the Ribosome

Evidence that snoRNA do more than modify nuclear RNAs comes from multiple lines of study, including human developmental genetics, chromatin studies and cancer biology. The genetic conditions Prader–Willi syndrome (PWS) and Angelman syndrome (AS) have long been key topics in human genetics as they result from deletions at the same imprinted locus, and differentially manifest according to parent of origin [18]. However, it turns out that they are more remarkable still thanks to the involvement of snoRNA. As with most conditions caused by genomic deletions, initial attempts to define the critical altered region suggested that multiple genes may contribute [18], yet, as further data reduced the region for PWS and AS, focus shifted to a snoRNA host gene called SNHG14 [19]. SNHG14 occupies a sub-locus called SNURF-SNURPN and encodes 47 copies of SNORD115, 29 copies of SNORD116 and a number of single copy SNORDs, e.g., SNORDs 64, 108, 109A and 109B [19]. In establishing which of the SNORDs was responsible, several mechanistic studies first pointed to the SNORD115 family [20,21]. Then later, Sahoo et al. examined patients with smaller deletions and concluded that loss of SNORD116 copies was the critical event [22,23,24]. Importantly, mouse models were generated to test the effects of SNORD116 loss. Although the phenotypes were subtle, they recapitulated relevant changes in diet hormones and cognitive defects [25,26,27,28], whereas a similar model for SNORD115 turned out to be less affected [29]. A later mouse study, specifically targeting deletion of SNORD116 to the hypothalamus, produced a more pronounced phenotype, including changes in body weight [30].

Despite the loss of SNORDs being firmly implicated, the mechanism for how this causes PWS and AS remains unclear, particularly since they lack complementarity to rRNAs [19]. Early work on SNORD115 suggested that it may alter splicing of mRNA encoding 5-hydroxytryptamine receptor 2C [29,31]. One of the few molecular studies in the SNORD116 knockout mice suggest that they have altered DNA methylation in neuronal genes during the diurnal cycle [32]. Therefore, the emerging picture for these SNORDs is that they are involved in functions quite distinct from the nucleolus. Similar findings are now being reported for orphan snoRNA with relevance to cancers.

Schubert et al. used immunoprecipitation studies to identify specific RNA species that are physically involved in chromatin compaction using drosophila cells. The molecules that were detected included many snoRNA [33]. Falaleeva et al. reported that SNORD27 could operate within a novel complex to compete with U1 snRNA RNPs (snRNPs) in order to alter splicing of mRNA encoding E2F Transcription Factor 7 (E2F7). E2F7 contributes to both head and neck cancers and retinal cancers [34]. Another study found the expression of SNORD88C was similarly altered mRNA splicing for fibroblast growth factor receptor 3 (FGFR3) [35], which contributes to the progression of multiple cancer types [36,37,38]. Interestingly, this mechanism appeared to involve shorter derived sdRNAs from SNORD88C, and as it turns out, this may be a common way that snoRNA expand their influence. Ender et al. analysed small RNAs associated with immunoprecipitated human AGO1, which forms part of the RISC complex involved in RNA silencing. They found that although the majority of identified species were miRNAs, a fraction were clearly derived from snoRNAs [39]. They went on to show that for each snoRNA represented, SCARNA15 was processed by Dicer and could regulate a reporter gene representing the 3′UTR of cyclin dependent kinase 19 (CDK19) [39]. Moreover, Kawaji et al. performed an unbiased sequencing of human small RNAs and found multiple classes of 20–40 nt species that originated from noncoding RNAs, including snoRNA [12]. This was later confirmed by other studies [13]. More recently, Kalantari et al. demonstrated that RISC complexes can operate both in the nucleus and cytoplasm, which allow sdRNAs to potentially form complexes at either site [40]. Taken together, these various studies show that snoRNA may be engaged in a wide range of molecular mechanisms.

## 4. Orphan SNORDs May Be Involved in Regulating Methylation of mRNA

Importantly, RNA methylation is now also recognised as a frequent modification of cytoplasmic mRNAs in addition to nuclear RNA [41]. In addition, several reports have shown this to be regulatory in nature [42,43,44,45] and significant to cancer progression and metastasis [46,47,48,49,50]. An important type of mRNA methylation is adenosine N6-methylation (m6A), which is catalysed by the methyltransferase-like proteins 3 and 14 (METTL3/METTL14) [51]. In localisation studies, METTL3 was found in the nucleolus alongside FBL, where it was demonstrated to co-modify rRNA [52]. Another type of mRNA methylation is ribose 2′-O-methylation (2′OMe). Elliot et al. used a knockout mouse for SNORD50A to test whether it altered 2′OMe modification of the mRNA for peroxidasin (Pxdn). The methylation was altered and it changed mRNA stability and translation [53]. Therefore, snoRNA may potentially contribute to cancer progression by supporting the methylation of mRNA.

## 5. SnoRNAs Are Important Markers of Cancer Establishment, Progression and Metastasis

Early clues that snoRNA contribute to cancer biology came from several sources. The condition dyskeratosis congenital carries an increased risk of cancers and is caused by mutations in genes involved with snoRNA nucleolus and Cajal body functions, including DKC1 that mediates pseudouridation [54]. SNHG5, a snoRNA host gene that harbours SNORD50, was mapped to the chromosome breakpoint t(3;6)(q27;q15) involved in human B-cell lymphomas [55]. Later, it was also found to be commonly deleted in prostate cancers [56]. Liao et al. used GeneChipR Oligo arrays to show SNORA42 act as an oncogene in non-small cell lung cancers (NSCLC) [57,58]. Similar work showed that SNORD78 also contributed to this disease [59]. Valleron et al. used a RT-qPCR approach to analyse a panel of 80 snoRNA in a subclass of T cell lymphomas. Here, they detected a strong association between SNORD71 and disease outcome [60].

As genome-wide expression analysis and RNA-Seq technologies have become more readily available, a number of cancer investigators have used them to assess snoRNA. Schulten et al. analysed gene expression in breast cancer metastases in the brain using microarrays and compared them to non-metastatic breast cancers. In addition to 13 protein coding genes, those differentially expressed included 6 SNORDs, 13 SNORAs and 1 SCARNA, suggesting that the snoRNA supported metastatic progression [61]. Gao et al. analysed 178 non-small cell lung cancers (NSCLC) using a combination of RNA-Seq and RT-qPCR. In total, 458 snoRNA were detected and 29 were elevated compared to normal tissues. In separate training and testing cohorts, they established a prognosis predictor based on three snoRNA: SNORA47, SNORA68 and SNORA78 [62]. Crea et al. performed RNA-Seq on xenografts generated with paired primary and metastases from prostate cancers to identify 21 snoRNA that correlated with metastasis. Here, they highlighted SNORA55 as a probable driver gene [63]. Then, in a landmark pan-cancer analysis, Gong et al. employed bioinformatic mining of small RNA data from the Cancer Genome Atlas (TCGA), representing 31 human cancers. They found approximately 400–500 snoRNA expressed in each cancer type, all of which were generally elevated in expression compared to normal tissues. In total, 203 were associated with the clinical stage and 355 had associations with patient survival, including 229 SNORDs, 108 SNORAs and 18 SCARNAs [64]. Then, focusing on smaller sdRNA sequences, Chow et al. undertook a similar pan-cancer analysis of the TCGA data and found that they too have clear relationships with clinical outcomes across cancers [65]. Previously, Uzunova et al. employed a combination of RNA-Seq and RT-qPCR in a cohort of 106 prostate cancers. They determined that sdRNAs arising from SNORD44, SNORD74, SNORD78 and SNORD81 were upregulated, with SNORD78 associating with metastatic disease [66]. Taken together, these various association studies suggest that snoRNA have significant prognostic value and are well-placed to be contribute to cancer progression and metastasis.

## 6. SnoRNA Host Genes May Independently Contribute to Cancer Progression

Concurrently, there has been a strong interest in the snoRNA host genes themselves and, in short, their expression is generally elevated in cancers and often prognostic [67]. However, it turns out that the snoRNA host gene mRNAs often retain introns and carry snoRNA sequences into the cytoplasm. Therefore, their true mechanisms of action require further clarification. GAS5 was cloned in 1988 from a cDNA library to represent growth-arrested NIH/3T3 mouse fibroblasts [68]. Only later was this recognised as a snoRNA host gene [69]. Its expression inhibits the growth of the human T lymphocyte CEM-C7 and MOLT-4 cell lines [70] and similar results have been reported for multiple other cell types [71,72,73,74,75]. ZFAS1, which encodes SNORD12, SNORD12A and SNORD12B, was also cloned early [76] and identified as being expressed in mouse mammary glands and breast cancers [77]. The knockdown of the gene promoted proliferation in breast cancer cells and was silenced in ductal carcinoma in situ (DCIS) [77]. However, subsequent work has shown ZFAS1 to have differing significance in other cancer types such as liver, lung, gastric and colorectal cancers, where it may instead promote tumour growth and metastasis [78,79,80,81]. Importantly, multiple studies have suggested that ZFAS1 operates in part as a decoy or “sponge” for miRNA species [78,80]. This concept has emerged as a common theme for the snoRNA host genes as a group [67]. However, it is an open question as to why the expression of snoRNA would be intrinsically linked with the sponging of miRNA. The expression of SNHG1 has been associated with worse outcomes. For example, in multiple cancers, it acts as a miRNA sponge and promotes epithelial-mesenchymal transition (EMT) [82,83,84,85]. SNHG3 increased cell progression and sponged miRNAs in osteosarcoma, colorectal and liver cancers [86,87,88]. SNHG5 promoted proliferation and migration and acted as a miRNA sponge in breast cancers, melanomas, leukaemia and gastric cancers [89,90,91,92]. This theme continues with similar findings reported for SNHG6, SNHG7, SNHG12, SNHG15, SNGG16 and SNHG20 [67,93,94].

Maternally expressed 8 (MEG8) resides within another imprinted locus DLK1-DIO3, which is responsible for a muscle phenotype in sheep [95] and at least two different conditions in humans [96,97]. MEG8 contains 9 copies of SNORD113 and 31 copies of SNORD114, and thus is comparable to the SNURF-SNURPN locus in nature. Similar to the other host genes, MEG8 promoted EMT in lung and pancreatic cancer cells [98,99], and correlated with poor prognosis in liver cancers [100]. Overall therefore, there is a clear theme that snoRNA host gene expression generally impacts cancer progression and metastasis, perhaps even more so than the snoRNAs linked to their expression.

## 7. The Importance of Ribosomes in Cancer Progression and the Role of mTOR Signalling

When considering how snoRNA may contribute to cancer progression, it is important not to underestimate their canonical role of rRNA modification. Ribosome function is pivotal to cell growth and is upregulated in cancers [101]. The mechanistic target of rapamycin (mTOR) signalling pathway functions to coordinate growth signals from activated Ras and other pathways with ribosome control. The pathway is a principal driver of lymphocyte expansion, and a key oncogenic pathway within cancers [102]. The activation of mTOR leads to an altered constitution of the ribosome, increasing eukaryotic translation initiation factor 4E (eIF4E) inclusion [103], as well as the selective translation of 5′-TOP mRNAs. 5′-TOP mRNAs get their name from an oligopyrimidine tract in their 5′UTRs that aids their recognition by the ribosome. Further, they encode many of the ribosomal proteins themselves [104,105]. An analysis of snoRNA host genes revealed that these too are usually 5′-TOP mRNAs, so in effect the snoRNAs are controlled by the mTOR pathway [106]. In line with this, GAS5 expression in T lymphocyte cell lines altered their sensitivity to rapamycin [107]. SNORA24 deleted liver cancer cells injected into mice, resulting in tumours that synergised with oncogenic RasG12V and altered ribosome activity [108]. Interestingly, in the clinic, the blockade of mTOR has proved particularly valuable for inhibiting lymphocyte-mediated immune responses (e.g., to prevent organ rejection) and for treating cancers of the kidney. Both of which, may have relevance to to anti-cancer immunity.

## 8. The Contribution of snoRNA to Cancer Immunity Has the Potential to Contribute to Disease Progression

**Figure 2:**
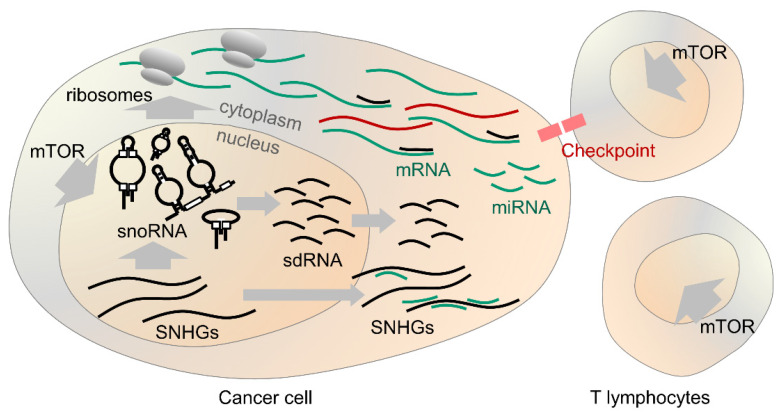
Overview of snoRNA in cancer. SnoRNA expression is promoted by mTOR both in cancer cells and T lymphocytes acting against them. SnoRNA facilitate the production of ribosomes and protein translation. SNHGs produce snoRNA and sponge miRNA in the cytoplasm. SnoRNA derived species may alter splicing and function of mRNAs. In some cases, snoRNA correlate with response to therapeutic targeting of the immune checkpoint proteins.

The ability of cancer cells to avoid detection and clearance by the immune system has recently become a central topic in oncology. Cancer cells upregulate expression of proteins (on both themselves and cells of immune system e.g. T lymphocytes) that normally protect non-cancerous cells from cytotoxic immune responses [109]. Antibody-based drugs targeting cytotoxic T-lymphocyte-associated protein 4 (CTLA-4), programmed cell death protein 1 (PD-1) and programmed cell death protein-ligand 1 (PD-L1) have proven superior to existing chemotherapy and radiation approaches for multiple cancer types [109]. In an analysis of acute myeloid leukaemias (AML) and acute lymphoid leukemias (ALL), together with a selection of normal white blood cell lineages, Warner et al. found significant differential expression of snoRNAs, both in the cancers and between the lineages from which they arose. Surprisingly, in addition to 30 SNORDs, 29 SNORAs and 4 SCARNAs from various genomic locations, the detected signatures were dominated by snoRNAs encoded in the SNURF-SNURPN and DLK1-DIO3 loci. Therefore, dysregulation of these imprinted sites appears to be a major feature of blood cancers and these data reiterate that snoRNA are important regulators of lymphocyte activity [110]. Of relevance, Zhong et al. reported that sdRNA derived from SNORD63 may control mRNA stability for lymphocyte regulator interleukin-4 (IL-4) [111]. In the pan-cancer sdRNA analysis conducted by Chow, clear associations were detected with PD-L1 expression for multiple cancer types and particularly for cancers of the kidney [65]. Finally, Motzer et al. recently defined molecular subtypes for kidney cancers (using all genes) and included measures of their response to anti-PD-L1 therapy. The 823 included cancers were grouped into seven subsets by non-hierarchal clustering. One of these was defined by marked snoRNA expression; this group was by far the most responsive to anti-PD-L1 [112]. Taken together, these findings suggest that snoRNA expression is of high relevance to lymphocyte function and the immune response against cancers, as well as the oncogenic mechanisms that promote their progression to metastatic disease (Figure 2).

## 9. SnoRNA Present Novel Therapeutic Opportunities to Combat Cancer Progression and Metastasis

Finally, given the emerging data that snoRNA are modifiers of cancer progression and metastasis, can they be therapeutically targeted? Several new technologies now provide significant hope [113]. Adenovirus-mediated delivery of SNORD44 from the growth inhibitory GAS5 gene inhibited CRC growth and synergised with rapamycin treatment in CRC cell line xenografts [114]. Antisense oligonucleotide (ASO)-mediated downregulation of SNORA23 reduced tumour growth, dissemination and liver metastasis in pancreatic ductal adenocarcinoma xenografts [115]. Therefore, snoRNA may have value both as drug targets in cancers or as therapeutics themselves.

## 10. Conclusions

Although snoRNA cancer research had a slow start, revelations from genome and transcriptome sequencing has now firmly illuminated their relevance to cancer biology. The extraordinarily large number of snoRNAs expressed in our cells, as well as the mounting evidence that they can alter cancer progression and patient survival, suggests that they have solid potential for therapeutic manipulation. Their control by mTOR and importance to ribosome function is a key aspect of their contribution, but their unknown functions are likely to be important also. Moreover, their specific contribution to lymphocyte function means that they may have relevance to a patient’s immunotherapy response. Emerging technologies that allow for their therapeutic manipulation makes them an important consideration for combating cancer progression and metastasis.

## Data Availability

Not applicable.
